# Reactive oxygen species: a volatile driver of field cancerization and metastasis

**DOI:** 10.1186/s12943-019-0961-y

**Published:** 2019-03-30

**Authors:** Zehuan Liao, Damien Chua, Nguan Soon Tan

**Affiliations:** 1 School of Biological Sciences, Nanyang Technological University Singapore, 60 Nanyang Drive, Singapore, 637551 Singapore; 2 Department of Microbiology, Tumor, and Cell Biology (MTC), Karolinska Institutet, Biomedicum, Solnavägen 9, SE-17177 Stockholm, Sweden; 3 Lee Kong Chian School of Medicine, Nanyang Technological University Singapore, 11 Mandalay Road, Singapore, 308232 Singapore

**Keywords:** Reactive oxygen species, Field cancerization, Metastasis, Tumor microenvironment, Cancer-associated fibroblasts, Tumor-associated macrophages

## Abstract

Field cancerization and metastasis are the leading causes for cancer recurrence and mortality in cancer patients. The formation of primary, secondary tumors or metastasis is greatly influenced by multifaceted tumor-stroma interactions, in which stromal components of the tumor microenvironment (TME) can affect the behavior of the cancer cells. Many studies have identified cytokines and growth factors as cell signaling molecules that aid cell to cell communication. However, the functional contribution of reactive oxygen species (ROS), a family of volatile chemicals, as communication molecules are less understood. Cancer cells and various tumor-associated stromal cells produce and secrete a copious amount of ROS into the TME. Intracellular ROS modulate cell signaling cascades that aid in the acquisition of several hallmarks of cancers. Extracellular ROS help to propagate, amplify, and effectively create a mutagenic and oncogenic field which facilitate the formation of multifoci tumors and act as a springboard for metastatic tumor cells. In this review, we summarize our current knowledge of ROS as atypical paracrine signaling molecules for field cancerization and metastasis. Field cancerization and metastasis are often discussed separately; we offer a model that placed these events with ROS as the focal instigating agent in a broader “seed-soil” hypothesis.

## Introduction

Worldwide, one in seven deaths is due to cancer; cancer causes more deaths than Acquired Immune Deficiency Syndrome, tuberculosis, and malaria combined. Recent statistics report estimates that there will be 18.1 million new cancer cases and 9.6 million cancer deaths worldwide in 2018 [1]. Current trends also suggest that cancer will remain as one of the leading causes of death and the most important barrier to increasing life expectancy globally. Cancer-related deaths have not rocketed because of significant advances in diagnosis. Improvements and a genuine postponement of death for various cancer patients are often due to better detection methods and not to better treatments [1, 2]. However, we have made less progress with traditional therapeutic options such as chemotherapy, radiotherapy, and surgery still dominates current anti-tumor treatment methods. Emerging therapeutic modalities such as chimeric antigen receptor T-cell (CAR-T) immunotherapy approach have proven to be very effective, but only a select subset of cancers responds to the treatment [3]. Furthermore, more than 90% of cancer deaths are caused by the metastatic spread of tumor cells from the primary to distant sites [4]. Yet, our understanding of this process is limited, and there are no specific therapeutic approaches to suppress cancer metastasis. Moreover, resistance to conventional chemotherapeutics and disease relapse remain persistent clinical challenges [4]. These observations imply an incomplete understanding of the cellular and biotic heterogeneity in the tumor.

Cancer is a genetic disease resulted from both internal factors (e.g., inherited mutations, immune conditions, hormones, etc.) and external factors (e.g., environment, diet, tobacco, diet, infection, radiation, among others) [2]. These factors can affect important genes such as proto-oncogenes, tumor suppressor genes and deoxyribonucleic acid (DNA) repair genes via cellular intermediates such as reactive oxygen species (ROS) [5]. ROS are major cellular intermediates. In most studies, ROS are used as an umbrella term to describe a heterogeneous group of cellular free radicals that contain oxygen (O_2_) derived from various intracellular processes and extracellular sources. ROS are highly reactive to biomolecules, and they can trigger multiple biological events [6]. ROS plays a contradictory role in cancer biology. Elevated ROS levels contribute to tumorigenesis, cancer progression and spreading via the promotion and maintenance of tumorigenic cell signaling which results in tumor cell proliferation, survival, autophagy, and metastasis [7]. In Table 1, we provide a non-exhaustive list of the various common ROS and their roles in cancer.

**Table 1:** ROS and Their Roles in Cancer

ROS	Roles in Cancer	References
Generic ROS	Activation of oncogenic Ras, Bcr-Abl, c-Myc which hyperactivates cell proliferation; induce Wnt/β-catenin pathway which increases metastatic potential; regulation of epithelial-mesenchymal transition (EMT) via matrix metalloproteinases (MMPs); regulation of nuclear factor kappa-light-chain-enhancer of activated B cells (NF-κB) pathways; contribution to drug resistance such as through high mutagenic rates	[7, 11]
Hydrogen Peroxide (H_2_O_2_)	Promotes phosphoinositide 3-kinases (PI3Ks)/RAC-alpha serine/threonine-protein kinase (Akt) survival pathway; induces mitogen-activated protein kinase (MAPK)/extracellular signal-related kinases (ERK) pro-proliferative signaling pathway; oxidative modification of phosphatase and tensin homolog (PTEN); oncogenic stabilization of hypoxia-inducible factor (HIF)-1α; conversion to hydroxyl radical	[35, 102, 103]
Superoxide (O_2_^•−^)	Conversion to H_2_O_2_, peroxynitrite; Stimulates AMPK activity to induce metastasis; oncogenic stabilization of HIF-1α	[102, 104]
Hydroxyl radical (•OH)	Initiates lipid peroxidation; promotes DNA mutagenesis	[105, 106]

Recently, the involvement of ROS as atypical context-dependent drivers of tumorigenesis is gaining attention [8]. On one hand, excessive ROS results in anti-tumorigenic effects via promoting cell death, inducing cell cycle arrest and senescence [9]. On the other hand, it is known that tumor cells promote their survival through enhanced ROS manipulation mechanisms, such as increased antioxidant levels or increased ROS production, to maintain the delicate balance in ROS level that supports their proliferation and survival [9, 10]. For example, autophagy can be induced by ROS to remove damaged mitochondria that contribute to oxidative stress, restoring ROS to physiological level [11]. However, the deletions of autophagy-related genes such as autophagy-related 5 (ATG5), autophagy-related 7 (ATG7) and beclin-1 (BECN1) can lead to defective autophagy. These deletions can result in the deregulated degradation of damaged mitochondria, and hence, elevated ROS production as well as oxidative stress [11].

Despite current knowledge in ROS signaling in cancer biology, the dual nature of ROS is still a huge conundrum in therapeutics targeting ROS. The inhibition or elevation of ROS levels can yield drastically different results [2, 12]. Recent discussions suggested dichotomizing the effects of ROS in cancer cells into two categories: early versus late stages. Depending on the stage of cancer progression, intracellular ROS plays a different role in cancer cell survival. At precancerous and early stages of cancer, intracellular ROS promote cancer initiation via inducing oxidative and base pair substitution mutations in pro-oncogenes such as Ras and tumor suppressor genes such as p53 [13]. Apart from inducing mutations, ROS can also modify site specific amino acids side chains which alter protein structure and functions [7]. Among the amino acids, cysteine (Cys) is more prone to oxidation by ROS due to the presence of thiol group. Cys appears to be the principal actor in redox signalling, functioning as a regulatory reversible molecular switch. As cancer progresses, the accumulation of excess intracellular ROS can trigger apoptosis, tumor cells escape apoptosis by producing high levels of intracellular antioxidants [13]. In the late stages of tumor evolution, metastatic tumors developed mechanisms that exploit ROS as a springboard for the dissemination of cancer cells. As a result, whether ROS play anti-tumor or oncogenic roles may depend on the different stages of cancer development and progression.

Many canonical pathways involved in tumor-promoting inflammation and cell proliferation have been shown to be activated by ROS. Transcription factor NF-κB plays an important role in cellular processes such as immune and inflammatory response, cellular proliferation and differentiation [14]. The canonical NF-kB pathway can be activated by oxidative stress and proinflammatory cytokines [15]. The activation of the canonical NF-κB pathway is dependent on the phosphorylation of IκB-Kinase (IKK) β, the ubiquitination-mediated degradation of NF-κB inhibitor alpha (IκBα), the translocation of NF-κB into the nucleus, resulting in the transcriptional activation of target genes [16, 17]. Studies have shown that ROS can trigger the activation of the NF-κB pathway via inducing the tyrosine phosphorylation of IκBα. IκBα, which is usually phosphorylated on serine-32 and -36 by IKK, undergoes ubiquitination and degradation for activation of the NF-κB pathway [18, 19]. Exogenous addition of H_2_O_2_ induces the phosphorylation of IκBα at tyrosine-42 as well as other tyrosine residues, resulting in the degradation of IκBα and the activation of the NF-κB pathway [17, 20].

Similarly, MAPK family which consists of ERK1/2, c-Jun N-terminal kinase (JNK), MAPK-11 and the MAPK1 pathway are important intracellular signal transduction pathways involved in cellular processes such as cell survival, cell death, growth, and differentiation [21]. Studies have demonstrated that ROS can activate the epidermal growth factor receptors (EGFR) and platelet-derived growth factor (PDGF) receptors without corresponding ligands. Thus, the activation of EGFR and PDGF can activate Ras and subsequently lead to the activation the ERK pathway [18]. Such modifications to the receptors conferred ligand-independent activation of the tyrosine kinase receptors and contribute to resistance against antibody-based therapies such as anti-EGFR (e.g. cetuximab, necitumumab) or anti-PDGF (e.g. Olaratumab). Furthermore, ROS may also activate the MAPK pathway via oxidative modification of intracellular downstream kinases such as apoptosis signal-regulating kinase 1 (ASK-1), a member of the mitogen-activated protein kinase kinase kinase (MAP3K) superfamily for JNK and MAPK-11 [22].

Another important pathway in cancer is the PI3K/PTEN pathway whereby several of the signaling mediators are redox sensitive and play important roles in field cancerization and metastasis [18]. ROS can act as signaling mediators by triggering oxidative modification of specific target molecules [23]. For example, PTEN can be modified by H_2_O_2_ via oxidation of the Cys thiol groups of phosphatases, resulting in its inactivation [7, 24].

Most studies on ROS in cancer were largely focused on the primary tumor. The roles of ROS in field cancerization and metastasis, which contribute to local and distant recurrence cancers, respectively, has been gathering attention. In the review, we will discuss the role of ROS in the tumor microenvironment (TME) in driving field cancerization and metastasis.

## Origin of ROS

ROS are produced by various biochemical and physiological oxidative processes in the cell. Mitochondria and nicotinamide adenine dinucleotide phosphate (NADPH) oxidase are the two major producers of ROS [25, 26]. ROS were once viewed merely as by-products of cell metabolism, but subsequent research showed that they have many roles in normal physiology. ROS serve as an important signaling molecule participating in a variety of cellular signaling pathways such as growth factor pathways, inflammation, engagement of integrins and adhesion to the extracellular matrix [27–30].

Oxidative stress contributes to aging and many diseases such as cancer, diabetes, and obesity. Oxidative stress occurs when excessive ROS accumulate in the cell due to an imbalance of oxidative and reductive activities, resulting in cellular damage. The antioxidant defense systems include superoxide dismutase (SOD), catalase, glutathione peroxide (GPx), glutathione reductase, glutathione S-transferase (GST), and glutathione, which are important to maintain a balanced level of intracellular ROS [31]. The NF-κB pathway can influence the ROS levels by increasing the expression of SOD, GPx and GST [18]. Mitochondria is an important source of intracellular ROS, such as superoxide O_2_^•−^ and H_2_O_2_ [26]. Intracellular H_2_O_2_ is formed by SOD-catalyzed dismutation from O_2_^•−^ generated within the mitochondrial matrix, intermembrane space, and outer membrane [26]. Increased ROS production in cancer cells can lead to elevated SOD expression as well as the inactivation of H_2_O_2_ scavenging enzymes, producing abnormally high levels of H_2_O_2_ [11].

**Fig. 1:**
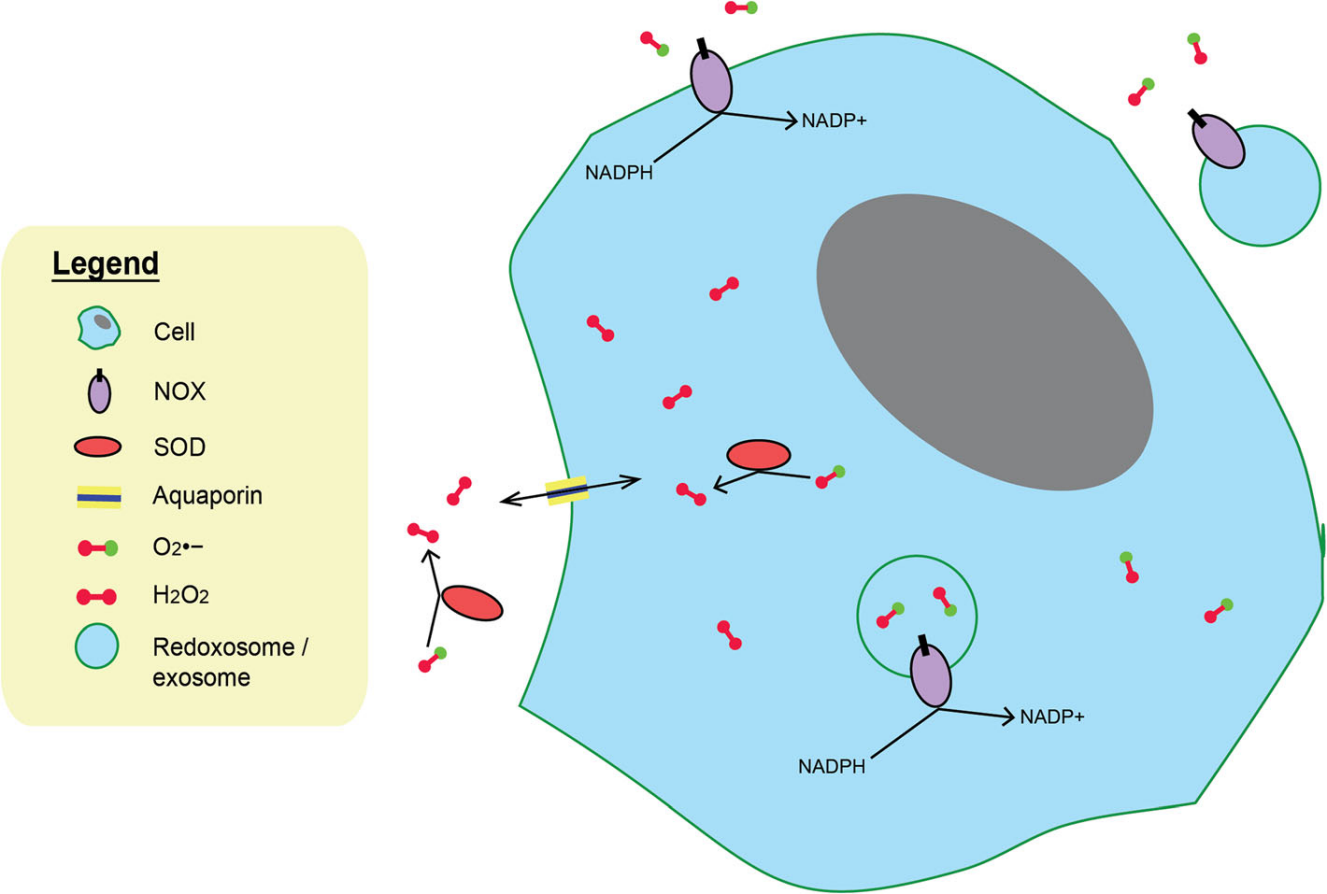
Fate of ROS: NOX proteins are integral membrane proteins of cells and release superoxides (O_2_^•−^) as products directly into the extracellular space. Functional NOX proteins can also be internalized into forming redoxosomes, producing superoxides (O_2_^•−^) within the redoxosomes. Furthermore, from cells such as macrophages, exosomal NOX complexes can be released and being incorporated into surrounding cells via endocytosis. Besides NOX, xanthine oxidase and nitric oxide synthase proteins (both not shown) can also generate extracellular and intracellular superoxides (O_2_^•−^) respectively. Superoxides (O_2_^•−^) are relatively impermeable through the cell membrane. However, intracellular and extracellular SOD proteins catalyze the dismutation of superoxides (O_2_^•−^) into H_2_O_2_ and O_2_. H_2_O_2_ molecules are relatively permeable through the aquaporins of the cell membrane and hence, can travel easily from cell to cell, providing regional oxidative stress

Apart from mitochondria, NADPH oxidase (NOX), an enzyme system, is a major source of extracellular ROS which mainly serves as communication molecules [2]. As integral membrane proteins, the expression of membrane-associated NOX releases O_2_^•−^ into the extracellular space [32]. Furthermore, these enzymes are also internalized to form redoxosomes, extending the intracellular reach of ROS [33]. Exosomes, containing functional NOX complexes to generate ROS, can also be released from cells such as macrophages into the extracellular space and transported to distant sites [34]. An elevated intracellular ROS inevitably results in a copious amount of extracellular ROS in the TME, thus neighboring normal cells will experience oxidative stress (Fig. 1) [35].

.

ROS have critical roles in tumor pathology. A high level of intracellular ROS due to defects in ROS production or detoxification processes can transform a normal cell into a malignant cell [2]. Indeed, cancer cells have elevated levels of intracellular ROS and extracellular ROS. Cancer cells have a high level of intracellular ROS due to reasons such as increased metabolic activity and mitochondrial energetics, alterations of the electron transport chain, expression of HIF-1 due to hypoxic condition and chronic inflammation [36]. Furthermore, cancer cells also have elevated expression of membrane-associated NOX [32]. Oncogenic KRAS was reported to increase the activity of NOX enzymes on the tumor cell membrane and hence, promote extracellular ROS generation [37]. While cancer cells may be more tolerant to oxidative stress via an elevated robust antioxidant defense, the consequence may be detrimental to the TME and the adjacent normal cells. For example, cancer cells expressed membrane-associated catalase to degrade extracellular ROS as well as express membrane-associated SOD to convert the more reactive O_2_^•−^ into reactive H_2_O_2_ [32, 33]. This might explain for the accumulation of oncogenic H_2_O_2_ in the TME, particularly in metastatic epithelial tumors [8, 35, 38].

Apart from cancer cells, various tumor-associated cell types also produce ROS and contribute to the oxidative microenvironment. Cancer-associated fibroblasts (CAFs) are one of the most abundant stromal cells in the TME and influence the pathology of cancer in many ways [39–41]. As CAFs is a cellular state rather than a cell type, CAFs are generally known as activated fibroblast in the TME with no precise molecular definition [42]. CAFs produce and are highly influenced by ROS [43]. CAFs have been shown to have an elevated level of H_2_O_2_ as compared to normal fibroblasts. The high production of intracellular and extracellular H_2_O_2_ by CAFs was due to impaired transforming growth factor beta (TGF-β) signaling [44, 45]. This impaired signaling leads to the suppression of the antioxidant enzyme GPx1 as well as the production of intracellular ROS by impaired mitochondrial function and extracellular ROS by induced NOX [46]. In addition, Caveolin-1, a negative regulator of NOX derived ROS, also increases the level of extracellular ROS production by CAFs [47]. Notably, normal fibroblasts treated with exogenous H_2_O_2_ or CAF-conditioned medium transformed into an oxidative, CAF-like state [35]. These newly transformed fibroblasts displayed elevated fibroblast activation protein (FAP) and α-smooth muscle actin (αSMA) expression levels, both of which are biomarkers of CAFs. Similar to CAFs, newly transformed fibroblasts became activated and desensitized to TGF-β. Normal fibroblasts treated with prolonged exogenous H_2_O_2_ displayed a significant increase in p65-NF-κB phosphorylation, triggering NF-κB activity. The NF-κB activation attenuated TGF-β signaling and hence, ensures the continued expression of FAP in the newly transformed fibroblasts [35].

In addition to CAFs, ROS are also released by many tumor-associated immune cells in the TME [48]. Tumor-associated macrophages (TAMs) in the TME mainly originate from blood-circulating monocytes that infiltrate into the TME and differentiate into mature pro-tumor macrophages mediated by cytokines in the TME [49–51]. Interestingly, the role of TAMs is a double-edged sword. As part of the immune system, macrophages are the first host cells to enter the TME and can potentially kill the cancer cells [52]. In vitro, activated macrophages showed anti-tumor activity via calreticulin binding receptors for cancer cell recognition [53]. Macrophages also display phagocytotic activity toward some damaged tumor cells [54]. However, the TME is known to have elevated levels of macrophages. During cancer initiation, cancer cells recruit macrophages via chemokines which amplify an inflammatory response. Macrophages also produce redoxosomes, i.e. exosomes containing functional NOX complexes into the TME, generating extracellular ROS and being incorporated into surrounding cells via endocytosis [34].

TAMs and CAFs are often detected close to each other, suggesting extensive communications and interactions between these two cell types [55]. The reciprocal relationship between TAMs and CAFs in the TME increases tumor malignancy, and ROS may be a key player in the interaction [56, 57]. ROS in the TME can trigger altered activation of macrophages and immunosuppression [58]. TAMs also release ROS which plays an essential role in immune alterations such as inducing apoptosis in lymphocytes [48, 59]. There was evidence indicating that the TME induces TAMs to activate immunosuppressive mechanisms via ROS production [60]. Macrophages exposed to increasing concentration of tumor fluid significantly increased intracellular ROS generation [58]. Elevated intracellular ROS corresponds to altered cellular redox homeostasis and oxidative stress [61].

Myeloid-derived suppressor cells (MDSCs) also promote cancer progression via ROS [62]. MDSCs are a heterogeneous population of cells which can suppress T cell responses and expand during inflammation and cancer [63]. These cells were first observed in patients with cancer [64–66]. Apart from their immunological functions, MDSCs were also reported to promote tumor angiogenesis, tumor cell invasion, and metastasis [67]. MDSCs are a unique component of the immune system which regulates the immune responses in cancer patients [63]. Indeed, up-regulation of ROS primarily by NOX is one of the major factors responsible for the immunosuppressive activity of MDSCs [68]. Granulocytic MDSCs, a subpopulation of MDSCs, were found to use ROS primarily as the mechanism of immunosuppression by close cell-cell contact with T cells [69, 70].

Although both TAMs and MDSCs were found to suppress T cell responses via different ROS mediated mechanisms, TAMs were the more potent immune suppressor [60]. MDSCs suppressed T cells via contact-independent H_2_O_2_ production and TAMs exerted their more potent immunosuppressive effects by the production of contact-dependent H_2_O_2_ [60, 69]. Certain subpopulations of MDSCs were found to be able to differentiate into immunosuppressive TAMs in the presence of tumor-derived factors or tumor-bearing hosts [69, 71, 72].

Hence, for cancer cells (the “seed”) to grow, expand and acquire more mutations to become malignant, a supportive TME (the “soil”) is required. ROS plays an essential role in creating the immunosuppressive “soil” ground for field cancerization and metastasis.

## ROS in field cancerization

Field cancerization was first reported in 1953 by Slaughter et al. and is often used to describe the development of abnormal epithelia and stroma bordering a tumorigenic area [73]. This posits the presence of a regional carcinogenic signal at these foci of cellular abnormalities, and if given enough time and exposure, the carcinogenic agent will cause irreversible changes to the cells leading to oncogenic transformation and field cancerization. Indeed, field cancerization results in multifocal primary tumors in proximity with a higher chance of recurrence even after resection of the malignant tumors [74]. Presently, field cancerization basically refers to pre-malignant changes in multiple and large areas of the primary tumor, within both the epithelial cells and surrounding stromal cells [75, 76]. Despite its clinical importance, only a few cytokines such as TGF-β, macrophage inhibitory cytokine 1 and PDGF-A have been implicated as possible field carcinogens [77–79].

Field cancerization can be initiated and propagated in many ways, including mutagen ROS [80]. The chronic exposure of high extracellular H_2_O_2_ promoted the transformation of normal epithelial cells and fibroblasts, indicating the presence of a premalignant field defect by oxidative stress in the TME [81–83]. H_2_O_2_ is an ideal field effect carcinogen due to its higher cellular plasma membrane permeability and longer half-life than other ROS counterparts [7, 35]. H_2_O_2_ aggravates cancer cell aggressiveness, transform primary epithelial cells by oxidative modification of the membrane associated PTEN and Src proteins, decreasing PTEN and increasing Src activities [8, 35, 43, 84]. Normal fibroblasts treated with H_2_O_2_ transformed into an oxidative, CAF-like state. In turn, these newly converted CAF-like cells produced higher H_2_O_2_ caused by an impaired TGF-β signaling [35]. These observations indicated that stromal cells, such as CAFs, engaged redox signaling circuitries and mitogenic signalings to reinforce their reciprocal relationship with the epithelial tumor, further supports that extracellular oxidative stress might act as a field effect carcinogen [35, 43]. Thus, ROS are atypical carcinogenic signals which promote stromal-mediated field cancerization [35].

**Fig. 2:**
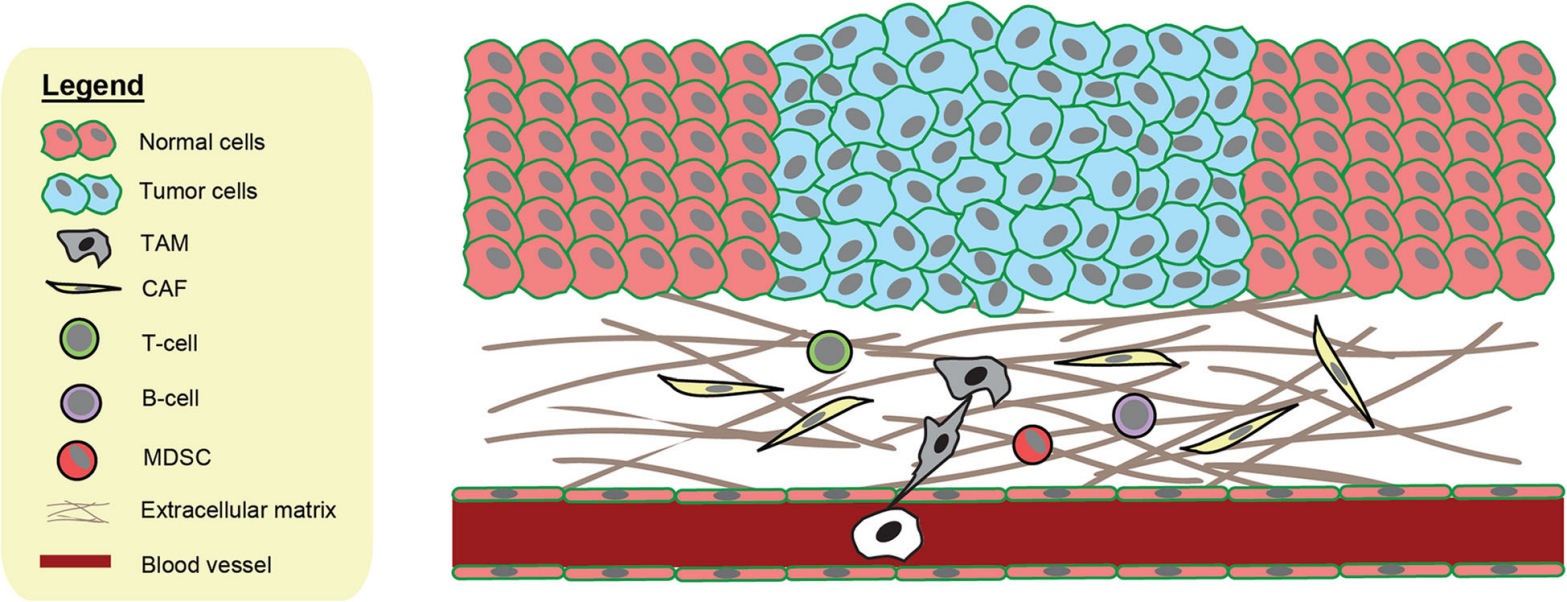
ROS in the TME: ROS can directly or indirectly modulate the functions of many cell types in the TME. ROS are able to transform normal epithelial cells and normal fibroblasts into malignant cells and CAFs respectively. ROS produced by cancer cells, TAMs and MDSCs can have an immunosuppressive effect on T-cells and B-cells. ROS can accumulate in the extracellular matrix, causing neighboring normal cells to acquire malignant phenotypes. Migration of the ROS-producing cells (cancer cells, CAFs, TAMs, MDSCs) to distant tissue or organ can start a new cancer field, transforming neighboring normal cells into cancer supporting cells or new malignant cells. This expanding field provides the appropriate “soil” for the survival and growth of newly-disseminated cancer cells or newly-transformed cells (the “seed”)

Conceivably, a single mutant cell represents the smallest origin capable of initiating field cancerization by causing neighboring normal cells to transform and to amplify an oxidative field [80]. Disseminated cancer cells can also start a new cancerized field by producing extracellular ROS. Although stromal cells such as CAFs do not transform into tumor cells themselves, alterations of stromal cells can promote field cancerization. This is because these stromal cells provide selective pressure such as oxidative stress in the field for particular newly mutated cells or disseminated cancer cells over the existing normal cells [80]. This provides the “soil” for newly mutated cells to acquire more mutations and progress towards cancer as well as newly migrated cancer cells to grow and survive in the new environment [80]. ROS in the new environment enact field cancerization by promoting oxidative stress, causing pre-malignant transformation of the surrounding cells as well as a series of immunosuppressive responses [80]. These findings suggest that the epithelial and stromal cells bathed in an oxidative milieu, continuously experienced oxidative stress that modulates their functions (Fig. 2) [35, 61].

Altogether, these results confirm the presence of stromal-mediated field cancerization, whereby the influences of stromal oxidative stress can be propagated and amplified, and effectively create a mutagenic or oncogenic field promoting multifocal tumor formations [35]. This finding features the mesenchymal-mesenchymal and epithelial-mesenchymal communications in the propagation of field effect and the creation of a TME niche [43].

## ROS in cancer metastasis

Metastasis is the spread of the primary tumor cells to distant organs, and this process is considered the main cause of cancer morbidity and mortality [4]. Once metastasis occurs, surgical excision of the primary tumor no longer guarantees disease-free survival, and the probability of cancer relapse in distal organs increases significantly. Metastasis is a complex, multi-step process beginning with cancer cells in the primary tumor undergoing EMT [85, 86]. This leads to an invasive tumor epithelial phenotype characterized by detachment from and degradation of the basement membrane [87]. Eventually, the invasive cancer cells gain access to local vasculature and/or lymphatics, intravasate and enter the systemic circulation. In the absence of basement membrane attachment, circulating cancer cells circumvent anoikis and evade the immune surveillance until they arrive at a secondary site where they extravasate and colonize distal organs [88]. Although most cancer deaths are the result of metastases, cancer research has mainly focused on the primary tumor.

Metastasis begins with EMT, which is a transdifferentiation program whereby epithelial cancer cells lose cell-cell adhesion and concomitantly acquire mesenchymal features of migration and invasion [86]. Several pieces of evidence have established a strong connection between EMT of epithelial cancer cells and ROS. TGF-β1 is well-established as one of the more prominent players of the induction of EMT [89]. TGF-β1 regulates urokinase-type plasminogen activator (uPA) and MMP9 to facilitate cell migration and invasion via the activation of NF-κB through the Rac1-NOXs-ROS-dependent mechanism [90]. Similarly, ROS also plays a crucial role in the regulation of EMT via the non-canonical TGF-β1-TGF-β-activated kinase 1 (TAK1) pathway. The increase in integrin:Rac-induced ROS by TAK1 deficiency results in a cascade of signals leading to accelerated EMT. Consistently, the expression of TAK1 was reduced in invasive squamous cell carcinoma (SCC), an observation absent from benign SCCs [91]. In a recent study, Matsuno and colleagues showed that ROS regulates EMT via the activation of nuclear factor (erythroid-derived 2)-like 2 (Nrf2), increasing Notch signaling which ultimately increases EMT [92]. It is known that exogenous ROS by sources such as ionizing radiation also results in the induction of TGF-β1 [93]. Taken together, increasing evidence revealed a multifaceted role of ROS in EMT. The fact that ROS is involved in several pathways that directly link to many critical EMT-inducing pathways underscores its importance and the crucial role of ROS in EMT.

Circulating cancer cells acquire anoikis resistance, where it loses its dependence on integrin-mediated extracellular matrix contact for survival and growth [38]. Many studies have shown that ROS is indeed one of the key players in anoikis sensitivity. The metastasis-associated gene, angiopoietin-like 4 (ANGPTL4) has been shown to be a key player. Via an outside-in signaling mechanism, ANGPTL4 protein engages with integrin to stimulate the production of ROS, which subsequently activates PI3K/Akt and ERK to confer anoikis resistance to tumor cells [38]. In a recent study, anoikis resistance in gastric cancer cells was attributed to an increase in NOX4-induced ROS generation [94]. The increase in ROS levels by NOX4 upregulates EGFR, which is a growth factor involved in cell survival and anoikis inhibition [95]. Similarly, another study also revealed that EGFR is directly associated with increased cell survival in the absence of extracellular matrix [96]. Indeed, intracellular ROS plays an integral role such as in the regulation of growth factors to bring about anoikis resistance of cancer cells which is an important step in metastasis.

In the final stage of successful metastasis, circulating cancer cells will extravasate and colonize the new secondary tumor site due to its predisposed microenvironment [97]. It has been revealed that interactions from primary tumor sites are able to set up a pre-metastatic niche in the secondary tumor site, and this determines the survival of disseminated tumor cell at the new site. The secondary TME also determines the outcome of the disseminated cancer cell, whether it thrives or remain dormant [98]. Increasing evidence has shown that ROS play a role in creating a ‘soil’ in distal organs, setting up a supportive tumor environment for disseminated cancer cells. One of such ways is through the cellular disposal of miR-23b via exosomes. miR-23b is a microRNA that is negatively involved in tumorigenesis through the regulation ROS [99]. The study also suggested that the transfer of exosome containing miR-23b from bone marrow mesenchymal stem cells, a common secondary tumor site in breast cancer, might enable a metastatic niche that promoted breast cancer cell dormancy, an observation that was contingent with breast cancer recurrence [100]. The accumulation of MDSCs from haemopoietic cells in the microenvironment of metastatic niches causes the increase in production of ROS that suppresses cytotoxic CD8^+^ T-cells activity, which promoted the disseminated cancer cell’s survival in the secondary tumor site [70, 101].

The involvement of ROS in various steps of metastasis makes it an integral player in the metastasis of tumors. The finding is important in guiding the way future clinical trials may be conducted as well as the development of redox-therapies that target the metastasis.

## Conclusion and perspectives

While field cancerization and metastasis are often discussed separately, these two phenomena may be analogously represented by a growing tree that eventually sprouts branches and develops a wide canopy. At its roots, a cancerized field fuels the acquisition of mutations or transcriptome changes, i.e., the “trunk” to promote growth. This co-evolution of tumor-stroma drives tumor cell clonal selection. Hence, some branches and offshoots begin to appear, i.e., intratumor heterogeneity. ROS helps to propagate, amplify, and effectively create a mutagenic and oncogenic field will facilitate the formation of multifoci tumors and act as a springboard for metastatic tumor cells. However, not all new profile change and mutations confer a selective advantage, and therefore some branches do not fully develop. Over time, tumor cells with the appropriate profile of metastatic “driver” genes within the cancerized field become aggressive and gain the capacity to invade, intravasate, evade the immune system and metastasize. The seed-soil concept by Paget becomes relevant in determining the survival of this disseminated tumor cells [97]. Again, ROS produced by exosomes could assist to interrogate and corrupt the distant soil for more effective colonization of the disseminated cancer cells. It is attempting to speculate that metastatic dormancy, where a disseminated tumor cell remains in a quiescent state at a remote organ while waiting for appropriate environmental conditions to begin proliferation again, may be partly attributed to poorly-prepared soil. Upon engraftment within a suitable secondary site, the metastasized tumor cell may once again enact field cancerization to corrupt its new microenvironment. ROS play important roles during field cancerization and metastasis, but many events remained relatively understudied. The scarcity of mouse models to monitor the production of volatile ROS by the tumor and to identify the cells affected by ROS in vivo remains a bottleneck to our understanding.

## Abbreviations

Akt: RAC-alpha serine/threonine-protein kinase
ANGPTL4: Angiopoietin-like 4
ASK-1: Apoptosis signal-regulating kinase 1
ATG5: Autophagy-related 5
ATG7: Autophagy-related 7
BECN1: Beclin-1
CAF: Cancer-associated fibroblast
CAR-T: Chimeric antigen receptor T-cell
Cys: Cysteine
DNA: Deoxyribonucleic acid
EGFR: Epidermal growth factor receptors
EMT: Epithelial-mesenchymal transition
ERK: Extracellular signal-related kinases
FAP: Fibroblast activation protein
GPx: Glutathione peroxide
GST: Glutathione S-transferase
HIF: Hypoxia-inducible factor
IKK: IκB-Kinase
IκBα: NF-κB inhibitor alpha
JNK: c-Jun N-terminal kinase
MAP3K: mitogen-activated protein kinase kinase kinase
MAPK: Mitogen-activated protein kinase
MDSC: Myeloid-derived suppressor cell
MMP: Matrix metalloproteinase
NADPH: Nicotinamide adenine dinucleotide phosphate
NF-κB: Nuclear factor kappa-light-chain-enhancer of activated B cells
NOX: NADPH oxidase
Nrf2: Nuclear factor (erythroid-derived 2)-like 2
PDGF: Platelet-derived growth factor
PI3K: Phosphoinositide 3-kinases
PTEN: Phosphatase and tensin homolog
ROS: Reactive oxygen species
SCC: Squamous cell carcinoma
SOD: Superoxide dismutase
TAK1: TGF-β-activated kinase 1
TAM: Tumor-associated macrophage
TGF-β: Transforming growth factor beta
TME: Tumor microenvironment
uPA: Urokinase-type plasminogen activator
αSMA: α-smooth muscle actin
